# Planned Venovenous-Extracorporeal Membrane Oxygenation as a Bridge to Orthotopic Liver Transplant Performed for Very Severe Hepatopulmonary Syndrome: A Case Report

**DOI:** 10.7759/cureus.63962

**Published:** 2024-07-06

**Authors:** Jefferson H Tyler, Vidyaratna Fleetwood, Ghassan Kamel, Divya R Verma, Govind Rangrass

**Affiliations:** 1 Anesthesiology and Critical Care, Saint Louis University School of Medicine, St. Louis, USA; 2 Abdominal Transplant Surgery, SSM Health Saint Louis University Hospital, St. Louis, USA; 3 Pulmonary, Critical Care, and Sleep Medicine, SSM Health Saint Louis University Hospital, St. Louis, USA; 4 Interventional Cardiology, SSM Health Saint Louis University Hospital, St. Louis, USA; 5 Anesthesiology and Critical Care, SSM Health Saint Louis University Hospital, St. Louis, USA

**Keywords:** pulmonary embolism, extracorporeal membrane oxygenation, perioperative management, orthotopic liver transplantation, hepatopulmonary syndrome

## Abstract

Concerns related to poor oxygenation in patients with severe hepatopulmonary syndrome (HPS) may be prohibitive when considering their candidacy for liver transplantation. Extracorporeal membrane oxygenation (ECMO) has been utilized in only a few case reports as a bridge to liver transplant in patients with severe respiratory failure. We report a case of a 66-year-old man with cirrhosis and very severe (arterial oxygen pressure (PaO2) < 50 mmHg) hepatopulmonary syndrome who underwent an orthotopic liver transplant with the planned use of venovenous-ECMO. Pre-transplant echocardiography demonstrated a small-trivial patent foramen ovale (PFO) but following the resolution of hepatopulmonary shunting after liver transplantation, the PFO size enlarged and contributed to a thromboembolic stroke. We conclude that well-selected patients with HPS could benefit from the use of planned venovenous-ECMO and that a small-trivial PFO seen in a patient with HPS may warrant intervention prior to transplantation.

## Introduction

Hepatopulmonary syndrome (HPS) is a complication of end-stage liver disease that occurs in as many as 30% of patients with cirrhotic disease [1]. In HPS, vasodilation, secondary to impaired hepatic clearance and increased release of nitric oxide and endothelin, leads to the formation of intrapulmonary shunts [2,3]. This shunting limits gas exchange, leading to hypoxia, the degree of which is independent of the severity of hepatic disease. Until 1998, HPS was a contraindication to liver transplant until it was observed that transplantation was associated with a reversal in intrapulmonary shunting, resolution of hypoxemia, and acceptable survival [4]. Given the lack of medical therapies for HPS, liver transplantation remains the only definitive cure [5,6].

Concerns related to perioperative oxygenation challenges may preclude listing patients with the most severe disease [6]. Prolonged ventilatory support places them at higher risk for pneumonia, deconditioning, and worse recovery. The use of extracorporeal membrane oxygenation therapy (ECMO) as a bridge to liver transplant and for perioperative rescue therapy, including patients with HPS, has previously been described [7-9].

## Case presentation

A 66-year-old man with very severe HPS (PaO_2_ 45 mmHg on room air, requiring 15L of O_2_ via a non-rebreather mask at home) secondary to cryptogenic cirrhosis presented for orthotopic liver transplant (OLT) evaluation. The patient’s past medical history included hypertension and hepatocellular carcinoma and his model for end-stage liver disease (MELD) and MELD-Na scores were both 13 at the time of transplant. His preoperative evaluation included transesophageal echocardiography (TEE) with a bubble study, demonstrating a predominantly extracardiac shunt, confirmed by the entry of the large majority of bubbles into the left atrium from the right pulmonary veins, best visualized in a pulmonary vein-focused-view, as shown in Video 1. A small-to-trivial intracardiac shunt or patent foramen ovale (PFO) could not be ruled out due to the significant extracardiac shunting. Right heart catheterization results were unremarkable. Pulmonary function tests (PFTs) and CT chest showed no signs of pre-existing pulmonary disease.

**Video 1:** Pre-transplant TEE with a bubble study, pulmonary vein-focused view TEE: transesophageal echocardiography

After extensive discussions regarding the patient’s candidacy and perioperative planning, we decided to use venovenous-ECMO as a bridge to transplant and the patient was listed with MELD exception points for both HCC and HPS. After organ procurement and placement on a normothermic perfusion pump, general anesthesia was induced in the catheterization laboratory uneventfully with vasopressors used to maintain a mean arterial pressure >65 mmHg. A baseline arterial blood gas obtained on 98% fraction of inspired oxygen (FiO_2_) was notable for PaO_2_ of 97 mmHg. In addition to standard monitors, bilateral radial arterial lines and a transesophageal echocardiography probe were placed.

Percutaneous cannulation was achieved with a 31 Fr x 28 cm - 18 Fr x 51 cm dual lumen venovenous-ECMO cannula placed via the right internal jugular vein. The tip of the cannula was positioned in the pulmonary artery. A 9 Fr x 20 cm multi-lumen access catheter was placed in the left internal jugular vein for central venous access and a 16 g x 20 cm single-lumen central line was placed in the right femoral vein. All venous access was obtained under fluoroscopic guidance. Because of the altered coagulation profile in cirrhotic patients, we empirically gave a single dose of 7500 units of heparin intravenously. After de-airing the circuit, venovenous-ECMO was initiated with a flow of 3L/min at 5200 RPM and a sweep of 3L/min, where deoxygenated blood was drained from the right atrium and returned into the pulmonary artery.

**Figure 1:**
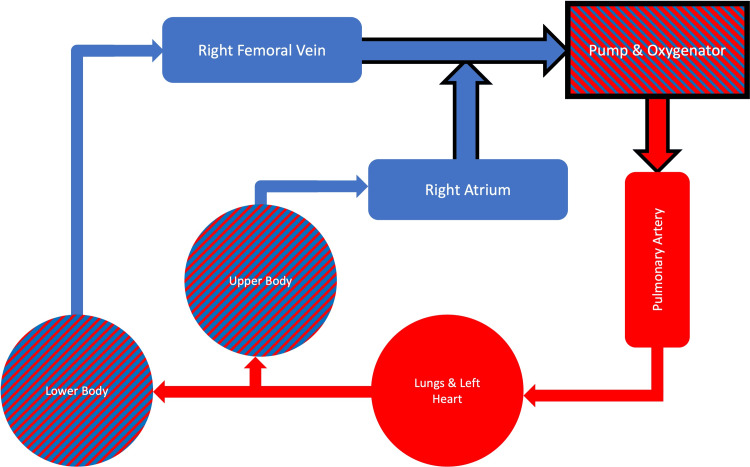
Venovenous bypass and extracorporeal membrane oxygenation (ECMO) circuit *Red*: oxygenated blood. *Blue*: deoxygenated blood. *Circles*: capillary systems. *Black lining*: artificial parts of the circuit. *Rounded boxes*: patient vasculature. *Sharp box*: ECMO circuit components. Blood flows in a clockwise manner. Image Credit: JH Tyler

The patient was then transported to the operating room, where the central line in the right femoral vein was upsized to a 17 Fr cannula, at which point a venovenous bypass was initiated using a Y-connector to merge the right femoral vein drainage cannula with the venovenous-ECMO circuit (Figure 1).

The heat exchanger on the bypass circuit was used to maintain normothermia. Prior to hepatectomy, a Hoffman clamp was placed on the tubing draining blood from the right atrium to the oxygenator to draw more flow from the femoral vein bypass cannula, which we were able to quantify using a flowmeter. After clamping of the infrahepatic and suprahepatic cavae, the donor's liver was sewn in using the bicaval technique. During this time, the total flow was 3.2 L/min, including 1 L/min from the femoral bypass cannula. The total anhepatic time was 73 minutes. The patient remained stable through reperfusion of the donor's liver and was empirically given sodium bicarbonate and calcium chloride to treat the expected metabolic derangements associated with reperfusion. During reperfusion, the flow was manually increased to 5 L/min to match the expected increase in preload. Following reperfusion, lower doses of vasopressors were required to maintain adequate blood pressure, and the femoral vein was decannulated. Anastomosis and closure were performed without incident. Estimated blood loss was 3,000 mL, with a urine output of 2,000 mL. The patient received 4,500 mL of 5% albumin, 3,150 mL of crystalloid, 9 units of fresh frozen plasma, and 8 units of packed red blood cells intraoperatively.

After surgery, the patient was transported to the intensive care unit (ICU) and intubated with venovenous-ECMO. The patient tolerated weaning of vasoactive support agents by post-transplant day (PTD) one. The immediate postoperative course was complicated by bleeding, which required re-exploration, repair, and washout on PTD three; given that the patient was expected to require prolonged respiratory support, a decision was made to pursue early tracheostomy, which occurred on PTD six; a bile duct stricture was treated with a stent on PTD seven; a right-sided pleural effusion required chest tube placement on PTD 10 (removed PTD 15); delirium; and Serratia marcescens pneumonia diagnosed on PTD 19 was treated with seven days of IV ceftriaxone.

After passing our institutional ECMO weaning protocol, he was decannulated on PTD 11. Ventilator requirements were weaned over the next several days, and the patient was tolerating supplemental oxygen via tracheostomy collar during the day and pressure support ventilation overnight by PTD 22. He was discharged to a long-term acute care hospital (LTACH) on PTD 28. While at the LTACH, the patient was weaned to a low-flow nasal cannula.

**Figure 2:**
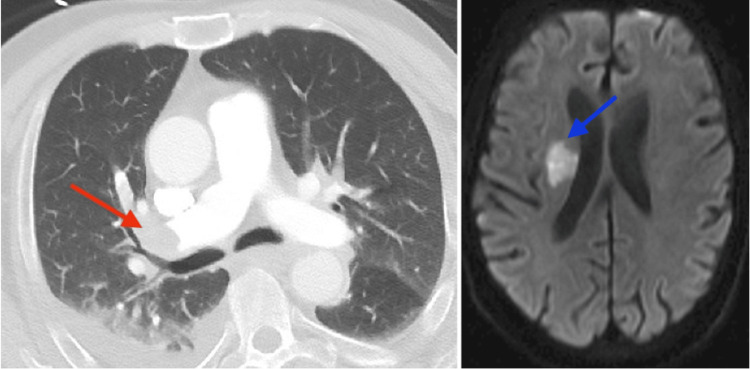
Results of imaging from code stroke *Left*: Computed tomography pulmonary embolus protocol. The embolus is shown by the red arrow. *Right*: Diffusion-weighted magnetic resonance imaging of the brain. Ischemic infarct is shown by the blue arrow.

On PTD 36, the patient presented to the emergency department from LTACH with sudden shortness of breath; left-sided weakness and facial droop; and altered mental status. The patient was found to have an ischemic stroke in the subcortical right middle cerebral artery territory and submassive bilateral pulmonary emboli (PE) with resulting right heart strain. Figure 2 shows the imaging performed at that time. Percutaneous thrombectomy of the PE was attempted but abandoned due to significant intracardiac right to left shunting.

The patient was then started on systemic anticoagulation with continuous intravenous heparin and readmitted to the ICU. Pulmonary embolism and stroke workup revealed an acute deep vein thrombus of the left profunda vein and significant enlargement of the PFO with significant right to left shunting (Video 2). The patient was weaned back to a low-flow nasal cannula and transferred to the floor five days after the stroke. At 12 months post-transplant, the patient is doing well with no residual neurological deficits and no need for oxygen support, and the PFO was subsequently closed percutaneously.

**Video 2:** Post-transplant TEE with bubble study, bi-atrial view TEE: transesophageal echocardiography

## Discussion

This case represents a novel use of ECMO to proactively manage perioperative complications related to hypoxemia in a patient with very severe HPS undergoing OLT. There were several unique aspects to the management of our use of ECMO. By utilizing a dual lumen right internal jugular vein cannula, the ECMO circuit operated through a single insertion site to achieve drainage of deoxygenated blood from the right atrium and return of oxygenated blood to the pulmonary artery. In the operating room, it gave us the ability to perform this operation on venovenous bypass by merging the right femoral vein bypass cannula with the ECMO circuit. Furthermore, it served as a right ventricular assist device during reperfusion, thus maintaining left ventricular preload, and should the patient have developed right ventricular dysfunction, it would have helped decrease congestion in the newly transplanted liver. Given that the patient had an estimated 3L blood loss intraoperatively and required ongoing resuscitation postoperatively requiring exploration for surgical bleeding, the support provided by the ECMO circuit avoided hypoxic insults to the new graft caused by pre-existing intrapulmonary shunts. The ECMO circuit requiring a single insertion site, allowing for easier postoperative patient transportation and early rehabilitation and mobility are important factors in decreasing ICU stays post-transplant [10,11].

Interestingly, postoperative changes in the patient’s physiology likely contributed to the stroke. As the intrapulmonary shunts closed, right-sided heart pressures increased, causing his previously insignificant PFO to become larger and permitting venous embolism to the brain.

Although this patient recovered with minimal lasting neurological deficits, we identified several strategies to potentially modify stroke risk for future patients. Some options include screening of PFOs via serial echocardiography postoperatively or prophylactic closure of PFO. Evaluating for intracardiac shunts can be especially difficult in patients with HPS given the degree of extracardiac shunting in these patients, as small shunts cannot be ruled out [12]. Although prior studies show no increased risk of cerebrovascular events in OLT recipients with PFO when compared to those without PFO, HPS patients may have altered stroke risk profiles, especially considering the increase in right heart pressures post-transplant [13]. Venous thromboembolism prevention measures that could be used in the future include prophylactic anticoagulation, preemptive placement of a permanent inferior vena cava filter, or asymptomatic screening for DVT.

## Conclusions

We report a case of a patient with very severe HPS undergoing OLT with planned venovenous-ECMO. This case describes the specific intraoperative management and feasibility of planned venovenous-ECMO, which may be an underutilized tool for safely performing OLT in patients with severe or very severe HPS. The preemptive use of venovenous-ECMO may be considered by transplant centers for patients with HPS. Additionally, this patient's postoperative course demonstrates that stroke in the setting of a pre-existing PFO is a major concern for HPS patients, as patients present to surgery with a large extracardiac shunt but may subsequently develop significant right-to-left intracardiac shunting following transplant, with the resolution of severe hepatopulmonary syndrome. Surveillance echocardiography post-transplant may be warranted to better monitor pre-existing PFOs to guide subsequent management and prevent thromboembolic stroke complications in post-transplant patients with HPS.
